# Plant-Derived Products for Treatment of Vascular Intima Hyperplasia Selectively Inhibit Vascular Smooth Muscle Cell Functions

**DOI:** 10.1155/2018/3549312

**Published:** 2018-10-11

**Authors:** Kang Xu, Mohanad Kh Al-ani, Xin Pan, Qingjia Chi, Nianguo Dong, Xuefeng Qiu

**Affiliations:** ^1^Department of Cardiovascular Surgery, Union Hospital, Tongji Medical College, Huazhong University of Science and Technology, Wuhan 430022, China; ^2^Tikrit University, College of Medicine, Department of Microbiology, P.O. Box 45, Salahaddin Province, Tikrit, Iraq; ^3^National Innovation and Attracting Talents “111” Base, Key Laboratory of Biorheological Science and Technology, Ministry of Education, College of Bioengineering, Chongqing University, Chongqing 400030, China; ^4^National Demonstration Center for Experimental Ethnopharmacology Education, College of Pharmacy, South-Central University for Nationalities, Wuhan 430074, China; ^5^Department of Mechanics and Engineering Structure, Hubei Key Laboratory of Theory and Application of Advanced Materials Mechanics, Wuhan University of Technology, China

## Abstract

Natural products are used widely for preventing intimal hyperplasia (IH), a common cardiovascular disease. Four different cells initiate and progress IH, namely, vascular smooth muscle, adventitial and endothelial cells, and circulation or bone marrow-derived cells. Vascular smooth muscle cells (VSMCs) play a critical role in initiation and development of intimal thickening and formation of neointimal hyperplasia. In this review, we describe the different originating cells involved in vascular IH and emphasize the effect of different natural products on inhibiting abnormal cellular functions, such as VSMC proliferation and migration. We further present a classification for the different natural products like phenols, flavonoids, terpenes, and alkaloids that suppress VSMC growth. Abnormal VSMC physiology involves disturbance in MAPKs, PI3K/AKT, JAK-STAT, FAK, and NF-*κ*B signal pathways. Most of the natural isolate studies have revealed G1/S phase of cell cycle arrest, decreased ROS production, induced cell apoptosis, restrained migration, and downregulated collagen deposition. It is necessary to screen optimal drugs from natural sources that preferentially inhibit VSMC rather than vascular endothelial cell growth to prevent early IH, restenosis following graft implantation, and atherosclerotic diseases.

## 1. Introduction

Intimal hyperplasia (IH) is a fibroproliferative disorder observed in vascular pathogenesis particularly in vessel anastomotic stenosis, atherosclerosis, blockage of vessel grafts, angioplasty, and in-stent restenosis [1]. IH is characterized by enhanced cell migration, proliferation, and differentiation that cause narrowing of the tunica intima. Several cells are associated with initiation and progression of IH, namely, vascular smooth muscle cells (VSMCs) [2], vascular adventitial cells [3], vascular endothelial cells (VEC) [4], and circulating bone marrow-derived cells [5]. These cells have different origins but may contribute to IH formation. For example, endothelial cells may undergo endothelial-to-mesenchymal transition (EndMT) acquiring a fibroproliferative mesenchymal phenotype whereas adventitia-derived stem cells may migrate to the intimal lesion site and differentiate into fibroblasts. VSMCs play a critical role in the initiation and development of intimal thickening and formation of neointimal hyperplasia [6, 7].

**Figure 1:**
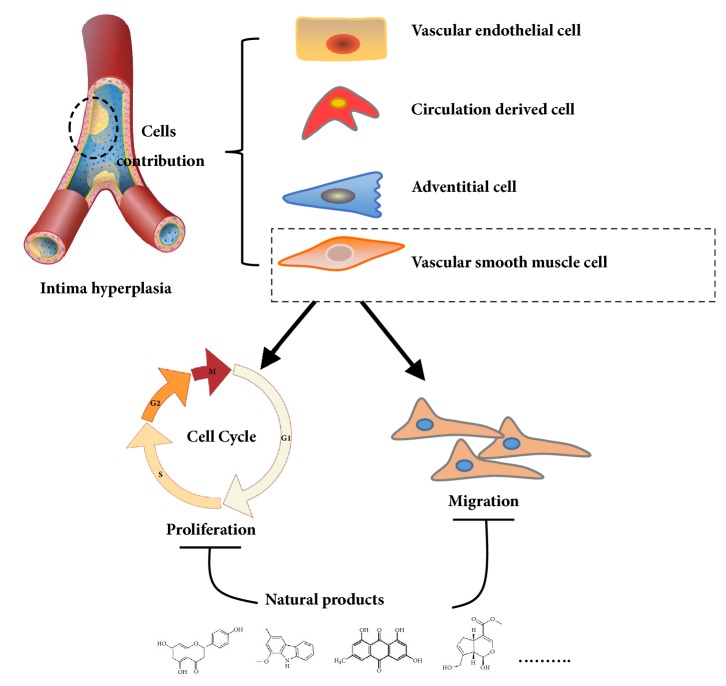
Graphic abstract for different natural compounds for inhibiting vascular smooth muscle cells proliferation and migration.

Many herbal medicines sourced from plants or foods have been used to prevent cardiovascular disease over the millennia. For example, green tea contains various flavanols that have antioxidative [8, 9], anti-inflammatory [10], antimicrobial [11], and hypolipidemic [9] effects. This pharmacological profile helps prevent atherosclerotic plaque formation caused by inflammation and oxidative stress. Red wine, another commonly enjoyed beverage, has long been believed to be rich in polyphenols [12], which act as powerful antioxidants. These assist in preventing lower density lipoprotein oxidation in heart disease and attenuating development of atherosclerotic disease in the hamster model [13, 14]. Resveratrol (3,5,4′-trihydroxy-*trans*-stilbene), a typical polyphenol extracted from red wine, has been proven to inhibit proliferation of VSMCs* in vitro* [15]. Many natural compounds have been reported to be active and to have potential utility as clinical medicines. Tanshinone is an isolate from* Salvia miltiorrhiza* that has been used against cardiovascular disease in China [16]. Therefore, many active compounds with cosmopolitan distribution are being used as herbal medicines or foods, giving hope for screening for potential therapeutic agents against IH (Figure 1).

Recent clinical studies have shown that rapamycin A, an VSMC inhibitor, prevents development of IH-induced vascular endothelial dysfunction [17]. This nonspecific cytotoxicity leads to stenosis and eventually to failure of vascular reconstruction after injury. Therefore, the ideal drug to prevent restenosis or IH is one that inhibits VSMC proliferation selectively while having minimal inhibitory effect on VEC proliferation.

## 2. Diverse Cells Involved in Vascular IH

**Figure 2:**
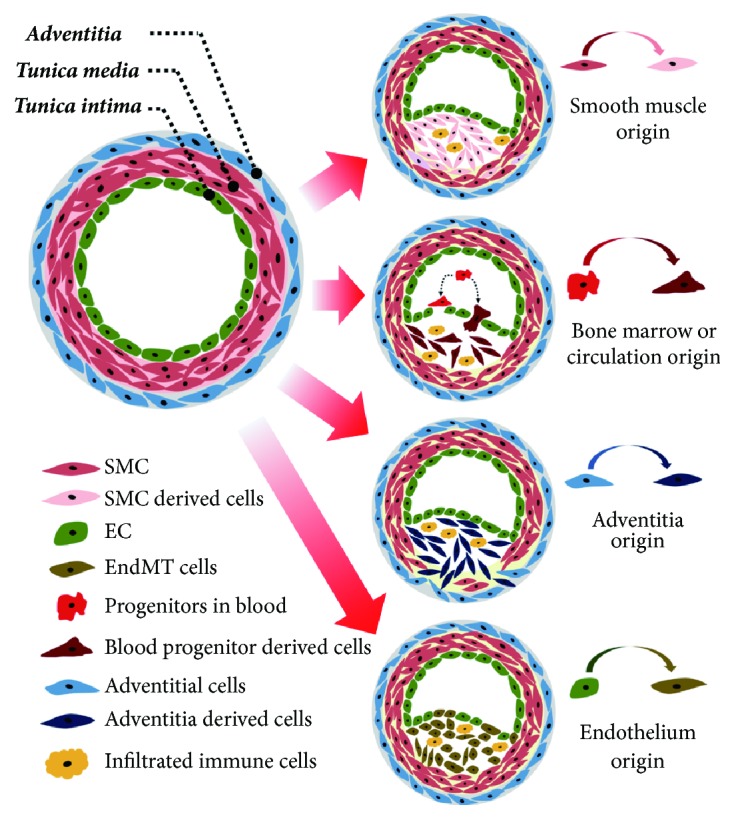
Four different cell origins contribute to blood vessel stenosis.

As stated earlier, four different cell types are involved in the initiation and progression of IH. These are VSMCs, vascular adventitial cells, VECs, and circulating bone marrow-derived cells (Figure 2). VSMCs play a critical role in the initiation of intimal thickening and the formation neointimal hyperplasia. Physiologically VSMCs exist in two phenotypes, i.e., differentiated cells and proliferating cells, which are responsible for maintaining the homeostasis and function of vascular vessels [2, 6]. Stimulation by certain growth and inflammatory factors, such as platelet-derived growth factor, tumor necrosis factor-*α* (TNF-*α*), and thrombin, results in dedifferentiation into mature VSMCs [18, 19]. Mature and differentiated VSMCs exhibit loss of contractility and increased proliferation and expression of ECM protein and various cytokines. This phenomenon is responsible for intimal thickening leading to the neointimal hyperplasia formation that is observed in early-phase atherosclerosis. 

Endothelial-to-mesenchymal transition is a phenomenon where endothelial cells acquire a fibroproliferative mesenchymal phenotype through differential stimulation [20]. These transitioned endothelial cells mimic fibroblasts and have increased ECM production and migration capabilities. In vascular diseases, these transitioned endothelial cells can quickly migrate and differentiate into smooth muscle-like cells serving as a potential contributor to IH [21, 22]. EMT is reported to be modulated by shear stress in an ERK5-dependent manner, to contribute to neointimal hyperplasia, and to induce atherogenic differentiation [23]. In addition to adventitia-derived stem cells, circulating smooth muscle progenitor cells have also been implicated in the pathogenesis of neointimal hyperplasia [24] and in the recruitment of endothelial precursor cells after vascular trauma. The presence of bone marrow-derived cells in solid neointima or allograft lesions suggests their crucial involvement in lesion formation following vascular injury [5, 25]. Although various cells contribute to IH pathogenesis, smooth muscle cells are the main culprits in lesion formation. Therefore, therapeutic strategies that maintain VSMCs in a terminally differentiated state and inhibit their proliferation and migration can be useful in preventing neointimal hyperplasia.

## 3. Antiproliferation, Migration, and Cellular Functions of Abnormal VSMCs as a Target to Decrease Intimal Hyperplasia

VSMCs in the normal vascular tunica media express a range of smooth muscle cell markers including smooth muscle cell myosin heavy chain (MYH11), 22-kDa SMC lineage-restricted protein (SM22*α*/tagln), alpha smooth muscle actin (ACTA2), and smoothelin. VSMCs* in vitro* and in atherosclerosis undergo phenotypic switching with reduced expression of these markers, while increasing capacity for cell proliferation, migration, and secretion of various ECM proteins and cytokines. These phenotypic switches have long been considered of fundamental significance in IH progression.

Most studies investigating inhibition of VSMCs adopt drugs like rapamycin, sirolimus, or tacrolimus to induce VSMC apoptosis and cell cycle arrest at G1/S phase, suppress ROS production, inhibit VSMC migration, and downregulate collagen deposition. These approaches do not recover the mature VSMC immunophenotypes, but they do decrease neointimal formation and prevent stenosis following vascular injury. To investigate the anticellular function of drugs on VSMCs many models have been established* in vitro* and* in vivo*. For the* in vitro* experiments, inflammatory cytokines like TNF-*α* or some growth factors such as platelet-derived growth factor (PDGF) are used for inducing abnormal proliferation and migration of VSMCs. For the* in vivo* experiments, IH is usually induced using the vascular endothelial denudation model or carotid artery ligation injury.

Dietary supplements and traditional herbal medicines are complementary medication approaches used in every society and are widely used for preventing IH in Asia and in other developed countries [26]. Many herbal drugs and foods have been verified as suppressing abnormal VSMC growth and inhibiting intima formation. The positive effects of the herbal medicines and plants depend on their active natural compounds including phenols, flavonoids, terpenes, and alkaloids. These natural products are involved in different signaling pathways that regulate abnormal VMSCs to attenuate IH.

## 4. Typical Signal Pathways Involved in Growth and Physiology of VSMCs in IH Disease

**Figure 3:**
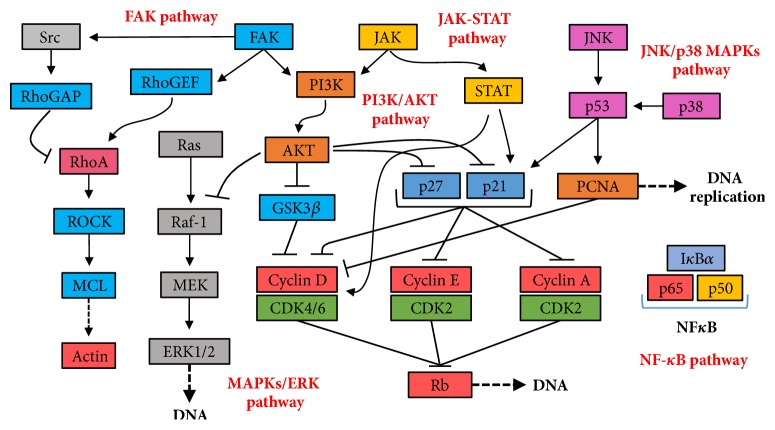
Key genes and pathways involved in restraining cell cycle and movements of VSMCs with natural products.

The six signaling pathways involved in most drug inhibitory VSMC studies (Figure 3) are mitogen-activated protein kinases/extracellular signal-regulated kinase (MAPKs/ERK), phosphatidylinositol 3-kinases/Akt (PI3K/Akt), Janus kinase-signal transducer and activator of transcription (JAK-STAT), focal adhesion kinase (FAK), and nuclear factor kappa-light-chain-enhancer of activated B (NF-*κ*B). MAPKs are involved in cell proliferation, differentiation, mitosis, cell survival, and apoptosis [27]. Three major families of MAPKs are extracellular signal-regulated kinase (ERK) [28], p38 kinase, and c-Jun N terminal kinase (JNK). These contribute to the two important signaling pathways, Ras/ERK-MAPK and JNK/p38-MAPK, which are involved in regulating VSMCs [29]. In antiproliferation studies of VSMCs, PI3K/Akt signaling pathway includes many key factors such as GSK3*β*, p21, and p27, which all inhibit cyclins and CDKs thereby interfering with cell cycle processes. GSK3*β* is one of the critical downstream molecules of the Akt signaling pathway involved in cell proliferation, metabolism, growth, and survival. It is reported that cyclin D is regulated by GSK3*β* [30] and that activation of GSK3*β* leads to exportation into cytoplasm for proteolysis, thus downregulating cyclin D1 expression [31]. The JAK-STAT signaling pathway transmits information from extracellular chemical signals to the nucleus resulting in DNA transcription and expression of genes involved in immunity, proliferation, differentiation, and apoptosis [32]. The downstream proteins in this pathway include cyclin D, p21, Bcl-2, and c-Myc, which are all directly involved in growth, apoptosis, and cell cycle progression in VSMC studies [33]. FAK is involved in cellular adhesion and migration [34]. FAK is typically located at structures known as focal adhesions, which are multiprotein structures including actin, filamin, and vinculin which link the ECM to the cytoplasmic cytoskeleton [35–37]. In addition, FAK interacts with PI3K and p53 [38, 39] and with the PI3K/Akt and MAPKs signaling pathways that are involved in cell cycle regulation. NF-*κ*B controls many genes involved with inflammation which are crucial to progression of diseases including arthritis, asthma, and atherosclerosis [40, 41]. Inflammation also mediates abnormal movement and growth of VSMCs, while suppressing inflammation could attenuate neointimal hyperplasia significantly [42–45]. Therefore, different signaling pathways are involved in VSMC inhibition, which provides preferential protein targets for future drug screening.

## 5. Different Natural Compounds Being Used for Preventing Neointimal Formation and Focus on VSMCs

### 5.1. Flavonoids Regulate Cell Cycle and Functions Inhibiting VSMCs Proliferation and Migration

Flavonoids are distributed throughout the plant kingdom and fulfill a diverse range of biological and pharmacological effects such as anti-inflammatory [46], antioxidant [47], antibacterial [48], antitumor [49], and antidiarrheal activities [50]. For treatment of cardiovascular disease, flavonoid studies have focused on reducing hypertension, risk of atherosclerosis, oxidative stress, and related signaling pathways in blood vessel cells, as well as modifying vascular inflammatory mechanisms [51, 52]. In this review, we described the chemical structure, category, source, and mechanism of action of some typical flavonoids that suppress VSMC function and inhibit IH (Table 1).

Nobiletin is widely distributed in citrus fruits and has been reported to inhibit VSMC proliferation and migration* in vitro* [44]. In addition, carotid balloon injured rats given nobiletin 25 mg/kg/day by gavage had significantly decreased neointimal hyperplasia via regulation of the ROS derived NF-*κ*B pathway and decreased serum TNF-*α* and IL-6 concentrations [44]. Cyanidin-3-O-glucoside, an anthocyanin flavonoid, inhibited TNF-*α*-induced NoxA1 (a type of NADPH oxidase) and downregulated expression of both TNF-*α* and NoxA1 at transcriptional and translational levels [53]. (2S)-Naringenin, a typical flavonoid isolated from* Typha angustata*, inhibited PDGF-BB-induced proliferation of VSMCs via a G0/G1 arrest by suppressing cyclin D1/E and CDK 2/4 [54]. Hu and colleagues found that icariin reduced the amount of ox-LDL-induced proliferation of VSMCs through suppression of PCNA expression and inactivation of ERK1/2 [55]. Puerarin, isolated from* Radix puerariae*, exerted inhibitory effects on high glucose-induced VSMC proliferation via interfering with PKC*β*2/Rac1-dependent ROS pathways, thus resulting in attenuation of neointimal formation [56]. Alpinetin is a well-known flavonoid isolated from a variety of plants such as* Alpinia katsumadai*,* Amomum subulatum*, and* Scutellaria rivularis*. It may have some protective effects on VSMCs as it decreases LDH leakage and inhibits production of NO in TNF-*α*-induced VSMC [57]. Hesperetin, a flavonoid, inhibits PDGFa-BB-induced pASMC proliferation via the AKT/GSK3*β* signaling pathway through upregulating p27 expression while suppressing cyclin D1/E, CDK2/4 and p38 [58]. Pinocembrin reduces the increased ERK1/2 phosphorylation that occurs in response to angiotensin II in both rat aortic rings* ex vivo *and VSMCs* in vitro* [59]. Glyceollins, which are isoflavonoids, inhibit PDGF-BB-induced hVSMC proliferation and migration by downregulating CDK2, cyclin D1, pPDGFr-*β*, phospholipase C*γ*1, Akt, and ERK1/2 and interfering with ROS generation, while upregulating p27^kip1^ and p53 expression levels [60]. Morelloflavone is a biflavonoid, which has been found to block injury-induced neointimal hyperplasia via inhibition of VSMC migration and downregulation of FAK, Src, ERK and RhoA expression [61]. Some studies have demonstrated that a natural flavonoid, kaempferol, may induce miR-21. This results in downregulation of ROCK4, 5, and 7, which are critical for cytoskeletal organization and cell motility, leading to decreased cell migration [62]. Finally, green tea is beneficial for health due to its antioxidant, anticarcinogenic, anti-inflammatory, and antiradiation effects [63–65]. A large number of flavonoids, especially flavan-3-ols (“catechins”), inhibit IH in a rat balloon injury model through upregulation of TIMP-2 expression to modulate MMP activity [66]. From the above review, flavonoids are an important candidate compound type for screening natural drugs capable of inhibiting VSMC growth.

### 5.2. Polyphenols as an Antioxidants Restrain VSMC Proliferation and Migration to Attenuate IH

Polyphenols are distributed widely in vegetables and plants, green tea, black tea, and red wine. Recent studies have shown that they possess antioxidant, anti-inflammatory, and cardioprotective effects [67–69]. Some typical polyphenols prevent IH by restraining VSMC function including proliferation, migration, and fibrosis (Table 2). Salvianolic acid B is a typical polyphenol that is usually isolated from* Salvia miltiorrhiza*. It markedly reduces neointimal thickness by inducing neointimal cell apoptosis through upregulating p53 expression levels [70]. In another study, salvianolic acid B protected hAECs and neointimal formation through inhibition of LDL oxidation by reducing ROS generation [71]. Magnesium lithospermate B, a derivative of salvianolic acid B, prevented diabetic atherosclerosis via the Nrf2-ARE-NQO1 transcriptional pathway [72]. Magnolol (a phenol) is a powerful antioxidant that inhibited balloon injury-induced rabbit IH by downregulating MCP-1 expression [73]. In another work, magnolol inhibited VSMC migration via the cytoskeletal remodeling pathway through inhibition of *β*1-integrin expression, phosphorylation of FAK and MLC20, and activation of RhoA and Cdc42 [74]. Lithospermic acid, a polyphenol, arrested cell cycle progression at the G1 phase via downregulating expression of cyclin D1 and inhibiting ROS generation and ERK1/2 phosphorylation [75]. Moreover, lithospermic acid attenuated LPS-induced VSMC migration by inhibiting MMP-9 expression in a dose-dependent manner (25-100 *μ*mol/L). Hispolon blocked balloon injury-induced neointimal hyperplasia via inhibition of VSMC proliferation. It also inhibited VSMC migration by lowering MMP-2/9 expression and increasing TIMP-1/2 expression through suppression of the FAK signaling pathway [76]. Lim and colleagues were of the view that obovatol blocked the cell cycle in G1 phase by downregulating expression of cyclins and CDKs, while selectively upregulating expression of p21^Cip1^, a well-known CDK inhibitor, both* in vitro* and* in vivo* [77].

Some studies have shown that curcumin (diarylheptanoid phenol) has potent antioxidant properties, which can be used for attenuating neointimal hyperplasia [78]. Curcumin has also been shown to inhibit PDGF-induced VSMC migration, proliferation, and collagen synthesis in a concentration-dependent manner [79], with a concentration range of 0.01 to 10 *µ*mol/L inhibiting VSMC proliferation and migration. Curcumin-coated stents inhibited neointimal formation in the rabbit iliac artery stent model. Moreover, curcumin inhibited LPS-induced MMP-2 activity in rat VSMCs through Ras/MEK1/2 and NF-*κ*B signaling [80].

Curcumin shows the ideal biological effects of inhibiting abnormal VSMC proliferation and migration without compromising VEC proliferation or delaying reendothelialization after blood vessel injury. Curcumin inhibited platelet adhesion to brain microvascular endothelial cells by decreasing expression of P-selectin, E-selectin, and GPIIb/GPIIIa in a concentration-dependent manner (30-240 *μ*mol/L). Curcumin antagonized the detrimental effect of rapamycin on aortic endothelial cells* in vitro,* through upregulation of eNOS [81]. Hence, curcumin very selectively inhibited abnormal VSMC functions, such as PDGF-induced proliferation or migration, without impairing VECs. As a result, curcumin has been regarded as an ideal drug for attenuating atherosclerosis and restenosis. In summary, polyphenols exhibit beneficial and wide ranging biological effects relevant to prevention of IH. Polyphenols are worthy candidate compounds to be screened as natural drugs for inhibiting VSMCs.

### 5.3. Terpenes Suppress Abnormal VSMC Function against Neointimal Formation

Terpenes are proven cell cycle inhibitors for various cell types, especially tumor cells [82, 83]. Like similar compounds with active sites for regulating VSMC mitosis and DNA synthesis, terpenes lead cell proliferation and function arrest via cell cycle blockade or apoptosis induction (Table 3). Betulinic acid, a typical terpene, has been reported to inhibit growth and proliferation of VSMCs via arresting G1/S cell cycle in a dose-dependent manner [84]. A monoterpene, (S)-(-)-perillic acid, has been reported to decrease protein prenylation leading to DNA synthesis and inhibition of VSMCs [85]. A sesquiterpene lactone, parthenolide, arrested VSMC G0/G1 cell cycle via upregulating p21 and p27. It also increased I*κ*B*α* expression and reduced Cox-2 expression in a time-dependent manner [86]. A special terpene, plumericin, arrested VSMCs in the G1/G0 phase of the cell cycle along with causing abrogated cyclin D1 expression, hindered Ser^807/811^-pRb protein [87], and blockade of STAT3 signaling via S-glutathionylation. Paclitaxel, a diterpenoid, has been used as an anticancer drug for decades and has been shown to prevent neointimal formation in oral administration studies [88]. Moreover, paclitaxel arrested VSMC G1/S phase by upregulating p21 and p53* in vitro* [89]. Epothilone D is a paclitaxel-like microtubule-stabilizing agent that was isolated originally from the myxobacterium* Sorangium cellulosum*. It inhibits neointimal hyperplasia through blockade of VSMC CDK2 and pRb [90]. *β*-Elemene protected VECs from injury induced by H_2_O_2_* in vitro* via downregulating MDA while upregulating T-AOC, SOD, GSH-Px, and CAT [91]. Meanwhile, *β*-elemene selectively inhibited VSMC proliferation/migration and inhibited neointimal formation* in vivo* following vascular injuries [91]. Recent studies have indicated that artemisinin effectively inhibited VSMC proliferation induced by TNF-*α* through apoptotic induction of the caspase pathway and cell cycle arrest [92, 93]. It also significantly inhibited neointimal formation in rat balloon injured carotid arteries. Therefore, terpenes are also notable candidate compounds for screening natural drugs capable of inhibiting VSMCs.

### 5.4. Alkaloids Exhibit Antiproliferation Biological Effect on VSMCs

Alkaloids are a group of naturally occurring chemical compounds that mostly contain basic nitrogen atoms. Alkaloids have diverse biological effects including those against tumors, hypertension, and pain. For vascular IH, some studies indicate that alkaloids hinder cell cycle progress, decrease ROS production, and inhibit VSMC migration (Table 4). A classic alkaloid, piperine, selectively inhibits VSMC proliferation with an IC50 of 11.8 *μ*mol/L without influencing VEC growth [94]. Coptisine was isolated from* Coptis chinensis* and suppresses VSMC proliferation selectively at lower concentrations with a GI_50_ of 3.3 *µ*mol/L (1.2 *µ*g/mL) [95]. Vinpocetine, a potential derivative of vincamine, inhibits high glucose-induced proliferation of VSMCs by preventing ROS generation and affecting MAPK, PI3K/Akt, and NF-кB signaling, Wang, Wen, Peng, Li, Zhuang, Lu, Liu, Li, Li, and Xu [96]. Vinpocetine arrested G1/S phase of the cell cycle by downregulating cyclin D1 and pERK1/2. Alongside these effects, vinpocetine also inhibited VSMC migration and ROS production [97]. A quinazolinone alkaloid, halofuginone, selectively inhibited cell proliferation, ECM deposition, and type I collagen synthesis in VSMCs versus VECs, which attenuated injury-induced IH [98]. Carbazole or murrayafoline A inhibited PDGF-BB induced abnormal proliferation of VSMCs by downregulating cyclin D1/E, CDK2/4, and PCNA and phosphorylation of Rb [99]. Review of these recent studies on the effects of alkaloids provides hope for identification of useful drugs capable of inhibiting VSMC growth and preventing IH.

### 5.5. Other Promising Natural Compounds for Preventing Intima Hyperplasia

As shown in Table 5, emodin is a typical anthraquinone compound beneficial for prevention of atherosclerosis due to its effects against inflammation, proliferation, and migration and its ability to induce apoptosis in VSMCs [100]. Moreover, emodin arrested growth and induced apoptosis and autophagy via enhanced ROS production and upregulation of p53 expression [101]. Emodin inhibited VSMC proliferation induced by angiotensin II through downregulation of PCNA and c-myc expression [102]. Moreover, emodin showed anti-inflammatory effects by inhibiting Hcy-induced CRP generation, a key inflammatory molecule in atherogenesis in VSMCs [103]. Emodin has also been shown to inhibit TNF-*α*-induced hASMC proliferation via caspase signaling and a mitochondrial-dependent apoptotic pathway by downregulating Bcl-2 and upregulating Bax expression [104]. Additionally, emodin reduced TNF-*α* induced migration of VSMCs by suppressing NF-*κ*B activation and MMP2/9 expression levels [105]. Our recent study demonstrated that emodin efficiently and concentration-dependently (0.05 to 5 *µ*mol/L) inhibited hVSMC proliferation more than hVEC proliferation* in vitro, *with less influence on reendothelialization of VECs in rat carotid artery balloon injury [106].

Methyl-protodioscin is a steroidal saponin that has been reported to inhibit neointimal formation by restraining VSMC proliferation and migration through downregulation of ADAM15, FAK, ERK, PI3K, Akt, and MMP-2/9 expression levels [107].* Salvia miltiorrhiza* has been used to prevent cardiovascular diseases in traditional Chinese medicine over the millennia. Tanshinone-IIA is a principal active component of* Salvia miltiorrhiza* that suppresses abnormal VSMC proliferation by cell cycle arrest at G0/G1 phase and inhibits phosphorylation of ERK1/2 and c-fos expression [108]. It has been reported that ajoene (1-50 *µ*nol/L) interfered with progression of the G1 phase in the cell cycle and restrained rat VSMC proliferation via inhibiting protein prenylation [109]. Gastrodin influenced the S phase entry of VSMCs and stabilized p27^KIP1^ expression. It also inhibited VSMC proliferation and attenuated neointimal hyperplasia by suppressing phosphorylation of ERK1/2, p38 MAPK, Akt, and GSK3*β* [110]. Genipin has been reported to inhibit TNF-*α* induced VSMC proliferation and migration in a dose-dependent manner by upregulating HO-1 expression, preventing ERK/MAPK and Akt phosphorylation, and additionally blocking generation of ROS [111]. Ginsenoside Rg1 is one of the main active components of* Panax ginseng* and is said to arrest G1/S phase in VSMCs by interfering with GRKs, PKC, and N-ras while upregulating p21 expression [112]. Vascular IH is significantly decreased when carotid artery balloon injured rats are intraperitoneally injected with ginsenoside Rg1 for 14 days [113]. Moreover, ginsenoside Rg1 significantly inhibited TNF-*α*-induced hASMC proliferation dose-dependently through downregulating cyclin D1, inactivating ERK1/2 and PKB, and upregulating expression of p53, p21^WAF/CIP1^, and p27^KIP1^ [114]. A coumarin called ostruthin is a major bioactive constituent of* Peucedanum ostruthium* and inhibited serum (10%)-induced VSMC proliferation in a dose-dependent manner [115].

Most foods contain various biologically active constituents that act to prevent and cure neointimal hyperplasia by inhibiting abnormal VSMC proliferation and migration. A well-known carotenoid, lycopene, is abundant in tomatoes and its products and has been reported to inhibit neointimal hyperplasia in a rabbit restenosis model. It does this by regulation of blood lipid concentrations and suppression of oxidative stress [116]. Sulforaphane, an organosulfur compound, mostly found in cruciferous vegetables significantly inhibited PDGF-BB-induced VSMC proliferation by upregulating p21 and p53 expression, while CDK2, cyclin E, and PCNA expression was suppressed [117].

## 6. Selective Inhibition of VSMCs versus VECs Shows Significant

**Figure 4:**
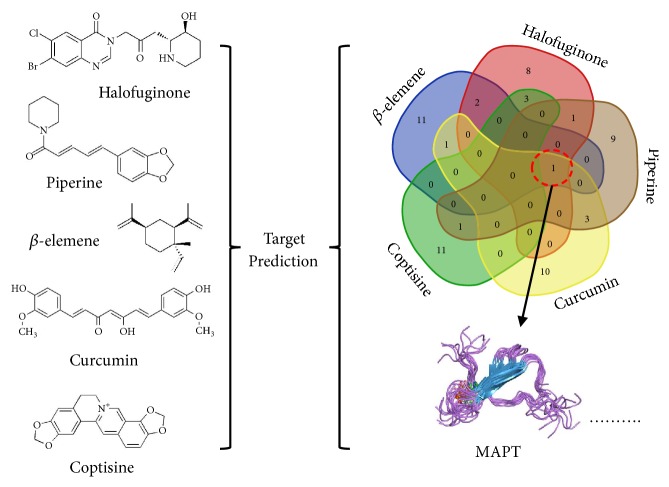
The compounds potential target: MAPT which is a common target.

Although many natural products inhibit VSMC function, most anti-smooth muscle proliferation drugs such as rapamycin (in-stent coating) also inhibit VEC proliferation and delay reendothelialization. This nonspecific cytotoxicity leads to restenosis and final graft or stent implantation failure. When screening for selective natural drugs that inhibit smooth muscle cell proliferation and migration, it is necessary to combine computer-aided design, bioinformatics, and a high-throughput screening platform. In this review, we selected certain drugs including chemosynthetic (idarubicin) and some natural (*β*-elemene, coptisine, halofuginone, piperine, and curcumin) compounds that possess specificity for suppressing proliferation of VSMCs over VECs. The chemical structure of the natural compounds has no typical similarity and cannot be analyzed using structural-activity relationships of molecular-protein binding sites. However, an online tool “Swiss Target Prediction” was used to predict potential targets of these compounds [118]. Most of the predicted targets of these drugs were membrane receptors, enzymes, kinases, proteases, or transporter proteins (Table 6). The analyses showed that microtubule-associated protein TAU (MAPT) is the most frequent protein target among them (Figure 4). This stabilizes microtubules and influences transportation of cellular secretory proteins. Moreover, MAPT has been reported to accelerate cancer cell growth [119], while its inactivation through gene knockdown suppressed cell proliferation [120]. Therefore, it is speculated that the diverse affinity of a natural drug to different functional protein targets may be one of the key factors for different selectivity profiles on VSMCs or VECs. Common targets like MAPT could be used as one of the important indicators in screening selective inhibitory drugs in future studies.

## 7. Conclusion

This review highlighted the originating four cells that may contribute to IH and then focused on VSMCs due to their involvement in intima formation as a consequence of abnormal proliferation, migration, and physiology. It further summarized typical signaling pathways such as MAPKs, PI3K/Akt, JAK-STAT, FAK, and NF-*κ*B and their involvement in the abnormal activities of VSMCs. Based on these the above cell origins and pathways, we organized and classified different natural isolates including phenols, flavonoids, terpenes, and alkaloids that have suppressing effects on VSMCs. In addition, many natural drugs not only induce apoptosis and arrest cell cycle in VSMCs, but also impair VECs leading to vascular restenosis and failure of blood vessel remodeling. Thus, it is crucial to screen desirable drugs from natural sources that preferentially inhibit VSMCs versus VECs to prevent IH in the early stages, restenosis following graft implantation, and even atherosclerotic diseases.

## Figures and Tables

**Table 1:** The structure, cells, category, source, and mechanism of typical flavonoid compounds on inhibiting VSMCs proliferation and migration.

**Compound name**	**Structure**	**Cells and animals**	**Category**	**Sources**	**Mechanism**
(2S)-naringenin	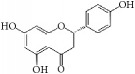	rASMCs	Flavonoid	*Typha angustata*	G0/G1 ↓; cyclins D1 ↓; cyclins E ↓; CDK2/4 ↓; PCNA ↓; pho of rb protein ↓
Catechins	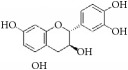	rASMCs and rat balloon injury	Flavonoid (Flavanols)	Green tea	TIMP-2 ↑, in vivo: TIMP-2 ↑
Icariin	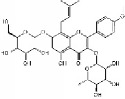	hASMCs	Flavonoid (Prenylated flavonol glycoside)	*Epimedium brevicornum*	pERK1/2 ↓; G1/S ↓; PCNA ↓
Morelloflavone	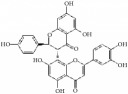	mVSMCs and mouse artery injury	Biflavonoid	*Garcinia dulcis*	FAK ↓; Src ↓; ERK ↓; RhoA ↓
Puerarin	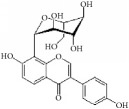	rASMCs and rat balloon injury	Isoflavone	*Radix puerariae*	ROS ↓; Nox ↓; PKC;PKC*β*2 ↓; Rac1 ↓; p47phox ↓; p67phox ↓
Kaempferol	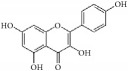	hpASMCs	Flavonoid	Widely (grapefruit, Ginkgo biloba)	miR-21 ↑; ROCK4/5/7 ↓
Nobiletin	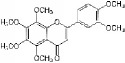	rASMCs and rat balloon injury	Flavonoid	Widely (citrus fruits)	ROS ↓; pERK1/2 ↓; NF-*κ*B p65 ↓, in vivo: TNF-*α* ↓; IL-6 ↓
Alpinetin	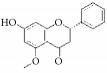	rASMCs	Flavonoid	Widely* (Alpinia katsumadai, Amomum subulatum, and etc.) *	LDH ↓; NO ↓
Cyanidin-3-O-glucoside	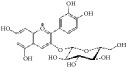	mASMCs	Flavonoid	*Hibiscus sabdariffa*	ROS ↓;NoxA1 ↓; pSTAT3 ↓
Hesperetin	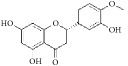	rpASMCs	Flavonoid	Widely (lemons and sweet oranges)	Block G1/S; cyclin D1 ↓; cyclin E ↓; CDK2/4 ↓; p38 ↓; p27 ↑; regulate AKT/GSK3*β* signaling pathway
Pinocembrin	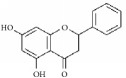	rAMSCs and rat aortic rings injury	Flavonoid	Propolis	ERK1/2 ↓; MLC2 ↓; AT1R ↓
Glyceollins	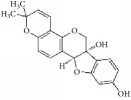	hASMCs	Isoflavone	Soybean	Arrest G1/S phase; CDK2 ↓; cyclin D1 ↓; p27kip1 ↑; p53 ↑; ROS ↓; pPDGFr-*β* ↓; phospholipase C*γ*1↓; Akt ↓; ERK1/2 ↓

**Table 2:** The structure, cells, category, source, and mechanism of typical polyphenols compounds on inhibiting VSMCs proliferation and migration.

**Compound name**	**Structure**	**Cells and animals**	**Category**	**Sources**	**Mechanism**
Salvianolic acid B	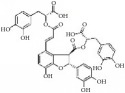	NeCs; HAECs and cholesterol-fed rabbits; rTASMCs and rats balloon injury	Polyphenol	*Salvia miltiorrhiza*	(1) p53 ↑; NeCs apoptosis, (2) ROS ↓; LDL oxidation ↓; lipid deposition ↓, (3) PCNA ↓; NQO1 ↓; via Nrf2-ARE-NQO1 pathway
Caffeic acid phenethyl ester (CAPE)	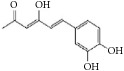	rASMCs	Polyphenol	*Honeybee propolis*	Blocking G0/1 to S phase; pp38 ↑;HiF1*α* ↑;HO-1 ↑
Hispolon	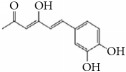	rTA-A10-VSMCs	Polyphenol	*Phellinus linteus*	MMP2 ↓;MMP9 ↓; TIMP-1 ↑;TIMP-2 ↑; pFAK ↓; pERK1/2 ↓;PI3K/AKT ↓
[6]-shogaol	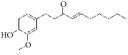	rASMCs	Phenols	*Zingiber officinale*	Inhibit DNA synthesis; activation of (Nrf2)/HO-1 pathway
Resveratrol	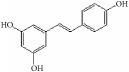	ncTASMCs; mASMCs	Polyphenol	Widely* (grapes, blueberries, raspberries, and etc.)*	c-Src ↓, Rac1 ↓, cdc42 ↓, IRS-1 ↓, MEKK1 ↓, MEKK4 ↓; p-Src; pFAK ↓; pAKT ↓; pERK1/2 ↓
Lithospermic acid	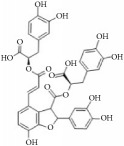	rTASMCs	Polyphenol	*Salvia miltiorrhiza*	ROS ↓; pERK1/2 ↓; cyclin D1 ↓; arresting cell cycle progression at the G1 phase; MMP9 ↓
Magnolol	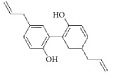	Cholesterol-fed rabbits; rVSMCs; rats balloon injury	Polyphenol	*Magnolia officinalis*	(1) MCP-1 ↓, (2) Reduce collagen type I deposition; *β*1-integrin ↓;pFAK ↓;pMLC20 ↓; RhoA ↓;Cdc42 ↓
Obovatol	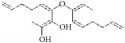	rASMCs; rats balloon injury	Biphenol	*Magnolia obovata*	Blocks the cell cycle in G1 phase; CDKs ↓;p21cip1 ↓
Curcumin	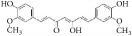	rTASMCs; rabbit artery injury; VECs; RAECs	Phenols	*Curcuma longa*	(1) Inhibits PDGF Receptor Binding; PDGFr ↓; pERK1/2 ↓; pAkt ↓, (2) P-selectin ↓; E-selectin ↓; GPIIb/GPIIIa ↓, (3) MMP2 ↓; pRas ↓; MEK1/2 ↓; NF-*κ*B p65 ↓, (4) Curcumin protects aortic endothelial cells; eNOS ↑; caveolin-1 ↓;

**Table 3:** The structure, cells, category, source, and mechanism of terpenes on inhibiting VSMCs abnormal proliferation, migration, and functions.

**Compound name**	**Structure**	**Cells and animals**	**Category**	**Sources**	**Mechanism**
Betulinic Acid	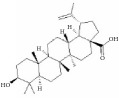	VSMCs	Terpene	Various plant sources widespread throughout the tropics	Inducing G1 Arrest and Apoptosis
Parthenolide	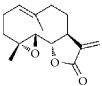	rVSMCs	Sesquiterpene lactone	*Tanacetum parthenium*	G0/G1 cell cycle arrest; p21 ↑; p27 ↑; I*κ*B*α* ↑;Cox-2 ↓
Plumericin	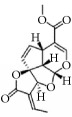	rAVSMCs	Iridoid (Terpene)	*Himatanthus sucuuba*	Block STAT3 signaling; arrest VSMCs in the G1/G0-phase; cyclin D1 ↓; pRb ↓
Paclitaxel	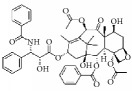	Rat balloon injury; hCASMCs (CC-2583) and VSMCs (CC-2571); rTASMCs and VECs	Diterpenoid	*Taxus cuspidata*	(1) prevent neointimal formation via oral administration, (2) arrest G1/S phase; p21 ↑; p53 ↑
Epothilone D	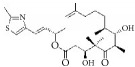	rTASMCs; carotid artery injury	Diterpenoid	*Sorangium cellulosum*	CDK2 ↓; pRb ↓
*β*-elemene	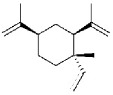	hUVECs and VSMCs (A7r5); rat balloon injury	Terpene	*Curcuma wenyujin*	Antioxidant; Casp 3/7/9 ↑; Migration ↓
Artemisinin	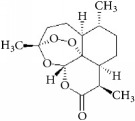	rVSMCs and rat balloon injury; rTASMCs	Sesquiterpene lactone	*Artemisia annua*	(1) arrest G0/G1 phase; cyclin D1/E ↓; CDK2/4 ↓; caspase 3/9 ↑; Bax ↑; Bcl-2 ↓, (2) PCNA ↓; caspase 3↑; Bax ↑; Bax/Bcl-2 ratio ↑
(S)-(-)-Perillic acid	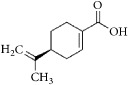	rASMCs	Monoterpene	Widely	Protein prenylation ↓

**Table 4:** The structure, cells, category, source, and mechanism of alkaloids on inhibiting VSMCs abnormal proliferation, migration, and functions.

**Compound name**	**Structure**	**Cells and animals**	**Category**	**Sources**	**Mechanism**
Piperine	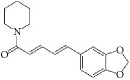	rASMCs	Alkaloid	*Piper nigrum*	Selectively inhibit VSMCs
Coptisine	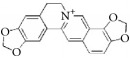	rVSMCs	Alkaloid	*Coptis chinensis*	Arrest G1/S phase
Vinpocetine	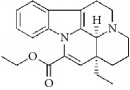	rVSMCs and rat balloon injury; rASMCs and mice carotid artery ligation injury	Alkaloid vincamine	Lesser periwinkle plants	(1) ROS ↓; apoptosis ↓; pAkt ↓; pJNK1/2 ↓; I*κ*B*α* ↓; PCNA ↓; cyclin D ↓; Bcl-2 ↓, (2) Arrest G1/S phase; cyclin D1 ↓; p27^Kip1^ ↑; inhibit migration; pERK1/2 ↓; ROS ↓
Halofuginone	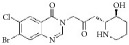	bASMCs	Quinazolinone alkaloid	*Dichroa febrifuga*	ECM synthesis and deposition ↓; Col I ↓
Murrayafoline A	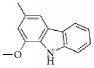	rASMCs	Carbazole alkaloid	*Glycosmis stenocarpa Guillaumin*	Arrest G1/S phase; cyclin D1/E ↓; CDK2/4 ↓; PCNA ↓; pRb ↓

**Table 5:** The structure, cells, category, source, and mechanism of promising compounds on suppressing VSMCs.

**Compound name**	**Structure**	**Cells and animals**	**Category**	**Sources**	**Mechanism**
Bilirubin	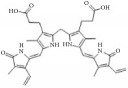	rVSMCs and mVSMCs; rat balloon injury	Ferric porphyrins	Heme	Inhibit MAPK signaling pathway; CDK2 ↓; Cyclin A/D1/E ↓; pRb ↓; YY1 ↓; p38 ↓
capsaicin	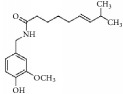	rASMCs	Capsaicinoids	Chili peppers	Inhibit DNA synthesis
Emodin	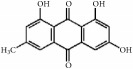	hUVSMCs; rTASMCs; hASMCs; rat balloon injury	Anthraquinone	*Rheum officinale*	(1) Arrest cell cycle, induce apoptosis and autophagy; ROS ↑; p53 ↑, (2) PCNA ↓; c-myc ↓, (3) CRP ↓;ROS ↓; pERK1/2 ↓; p38 ↓; PPAR*γ* ↑, (4) Induce apoptosis; Bcl-2 ↓; Bax ↑, (5) MMP2/9 ↓; NF-*κ*B activation ↓
Rhein	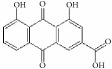	hASMCs	Anthraquinone	*Rheum palmatum*	Col I/III ↓; Wnt4/Dvl-1/*β*-catenin ↓; miR-126 ↑
Ajoene	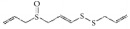	rASMCs	Organosulphur compound	*Allium sativum*	Inhibit protein prenylation and cholesterol biosynthesis
Gastrodin	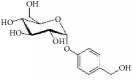	rASMCs, mice artery injury	Glucoside	*Gastrodia elata B1*	Block S-phase; stabilised p27Kip1; PCNA ↓; pERK1/2 ↓; pp38 ↓; pAkt ↓; pGSK3*β* ↓
Genipin	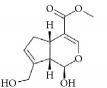	rTASMCs	Aglycon	*Gardenia jasminoides*	HO-1 ↑; pERK/MAPK ↓; pAkt ↓; ROS ↓
Ginsenoside Rg1	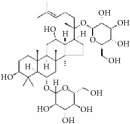	hASMCs; rat balloon injury	Steroid glycosides	*Panax ginseng*	(1) PCNA ↓; pERK2 ↓; c-fos ↓; MKP-1 ↑; (2) Arrest G1/S phase; GRKs ↓; PKC ↓; N-ras ↓; p21 ↑, (3) Cyclin D1 ↓; p53 ↑; p21WAF/CIP1 ↑; p27KIP1 ↑; inactivate PKB and ERK1/2
Ostruthin	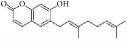	rTASMCs	Coumarins	*Peucedanum ostruthium *	Inhibit DNA synthesis
Lycopene	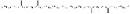	Rabbit artery injury	Carotenoid	Widely (tomatoes, red carrots,)	TG ↓; TC ↓; LDL-C ↓; HDL-C ↑; SOD ↑; T-AOC ↑; MDA ↓; PCNA ↓; pERK1/2 ↓; Nox1 ↓; p22^phox^ ↓; HMG-CoA ↓; ABCA1 ↑
Methyl Protodioscin	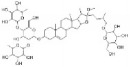	A7r5 VSMCs; rat balloon injury	Steroidal saponin	*Dioscorea collettii*	Arrest G1/S phase; ADAM15 ↓; MMP2/9 ↓; FAK ↓; ERK ↓; PI3K ↓; Akt ↓
Tanshinone IIA	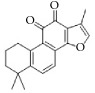	rASMCs; rat balloon injury	Phenolic acids	*Salvia miltiorrhiza*	Block cell cycle in G0/G1 phase; pERK1/2 ↓; c-fos ↓
Sulforaphane	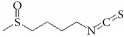	rASMCs; rat balloon injury	Organosulfur compounds	*Widely (cruciferous vegetables such as broccoli, Brussels sprouts, and cabbages)*	p21 ↑; p53 ↑; CDK2 ↓; Cyclin E ↓; PCNA ↓

**Table 6:** The selected potential targets of the compounds.

	**Idarubicin**	**Halofuginone**	**Piperine**	**β** **-elemene**	**Curcumin**	**Coptisine**
**Seq**	**Predicted target names (most related top 15)**					
1	MAPT	BCHE	MAOA	MAPT	MAPT	CHRM4
2	MBNL1	ACHE	MAOB	TDP1	TLR9	CHRM1
3	MBNL2	MAPK8	SIGMAR1	CXCR3	TDP1	CHRM2
4	MBNL3	MAPK9	MBNL1	SLC6A2	Unknown	CHRM5
5	MMP2	MAPK10	MBNL2	SLC6A3	MBNL1	CHRM3
6	MMP9	MAPK11	MBNL3	LDLR	MBNL2	BCHE
7	APP	MAPK14	MAPT	VLDLR	MBNL3	ADRA2A
8	SNCA	HTR1A	DRD2	LRP8	GLO1	CYP2D6
9	APLP2	HTR1B	DRD3	HSD11B1	AKT1	ADRA2B
10	SNCG	MAPT	HDAC3	BACE1	AKT2	ADRA2C
11	SNCB	HTR2A	HDAC1	HSD11B1L	AKT3	ACHE
12	TDP1	DRD2	HDAC2	BACE2	HSD17B3	HTR2A
13	EGFR	DRD1	DYRK1A	HTR1A	HSD17B12	HTR2C
14	ERBB2	OPRM1	HDAC6	HTR1D	CRYZ	HTR2B
15	ERBB3	OPRD1	CTSL1	HTR1B	APP	SIGMAR1

## Abbreviations

IH: Intimal hyperplasia
EndMT: Endothelial-to-mesenchymal transition
rASMCs: Rat aortic smooth muscle cells
rTASMCs: Rat thoracic aortic smooth muscle cells
VSMCs: Vascular smooth muscle cells
CA: Carotid artery
RAECs: Rat aortic endothelial cells
HAECs: Human aortic endothelial cells
VECs: Vascular endothelial cells
hUVECs: Human umbilical vein endothelial cells
hUVSMCs: Human umbilical vein smooth muscle cells
NeCs: Neointimal cells
rTA-A10-VSMCs: Rat thoracic aorta A10 vascular smooth muscle cells
ncTASMCs: Newborn calf thoracic aorta smooth muscle cells
mASMCs: Mice aortic smooth muscle cells
hPASMCs: Human pulmonary artery smooth muscle cells
rPASMCs: Rat pulmonary artery smooth muscle cells
hCASMCs: Human coronary artery smooth muscle cells
bASMCs: Bovine aortic smooth muscle cells
MYH11: Smooth muscle cell myosin heavy chain
SM22*α*/tagln: SMC lineage-restricted protein
ACTA2: Alpha smooth muscle actin
ECM: Extracellular matrix
TNF-*α*: Tumor necrosis factor-*α*
PDGF: Platelet-derived growth factor
ERK: Extracellular signal-regulated kinase
MMP: Matrix metalloproteinase
MAPK: Mitogen-activated protein kinase
JNK: c-Jun N terminal kinase
PCNA: Proliferating cell nuclear antigen
PI3K: Phosphatidylinositol-4,5-bisphosphate 3-kinase
AKT: Serine/threonine kinase 1
CDK: Cyclin-dependent kinase
JAK: Janus kinase
STAT: Signal transducer and activator of transcription protein
FAK: Focal adhesion kinase
NF-*κ*B: Nuclear factor kappa B
LDL: Low-density lipoprotein
ROS: Reactive oxygen specie
IL-1*β*: Interleukin 1-*β*
LPS: Lipopolysaccharide
Nox: NADPH oxidase
TIMP: Tissue inhibitors of metalloproteinase
NOS: Nitric oxide synthase
IC50: Half maximal inhibitory concentration
miR-21: MicroRNA-21
NO: Nitric oxide
LDH: Lactate dehydrogenase
eNOS: Nitric oxide synthase
pPDGFr-*β*: *β*-type platelet-derived growth factor receptor
ROCK: Rho-associated protein kinase
Rb: Retinoblastoma tumor suppressor protein family.
